# A Rare Case of Tracheal Amyloidosis in a Middle-Aged Person

**DOI:** 10.7759/cureus.85983

**Published:** 2025-06-14

**Authors:** Muhammad Ammar Iqbal, Danial Kaleem, Hira Gul, Junaid Zafar Sheikh, Umar Khan

**Affiliations:** 1 Respiratory Medicine, University Hospital Limerick, Limerick, IRL; 2 Medicine, University Hospital Limerick, Limerick, IRL

**Keywords:** endobronchial ultrasound, immunoglobulin light chain amyloid, lymphadenopathy, lymphoproliferative disorder, tracheal amyloidosis

## Abstract

Tracheal amyloidosis is a rare condition characterized by the extracellular deposition of misfolded proteins, primarily immunoglobulin (Ig) amyloid light chain (AL), within the tracheobronchial tree. Clinical manifestations include cough, exertional dyspnea, wheezing, hoarseness, chest tightness, and hemoptysis. Diagnosis involves imaging, such as CT of the neck and thorax, followed by histological confirmation via flexible bronchoscopy. Treatment is tailored to individual symptom severity. We present the case of a 71-year-old male patient who presented with progressive shortness of breath over six months. A CT thorax revealed mediastinal, subcarinal, and hilar lymphadenopathy, alongside high-grade stenosis of the left upper lobe bronchus. Endobronchial biopsy identified acellular material exhibiting apple-green birefringence under polarized light, consistent with amyloid deposition. Further excisional biopsy from the left inguinal region revealed a low-grade lymphoproliferative disorder with marked plasmacytic differentiation, contributing to AL amyloid production. The patient is currently receiving treatment for low-grade lymphoma under the supervision of a hematology consultant. This case highlights the diagnostic challenge posed by tracheal amyloidosis, emphasizing the importance of a multidisciplinary approach for effective diagnosis and management. Early recognition and comprehensive evaluation are essential for timely intervention, particularly in cases associated with underlying lymphoproliferative disorders.

## Introduction

Tracheal amyloidosis is a very rare entity characterized by amyloid deposition in the tracheobronchial tree. Symptoms vary according to the individual patient, but most of the symptoms are progressive shortness of breath, vague chest pain, cough, and seldom hemoptysis [1]. Depending on the location of amyloid deposition, it can also cause an airway obstruction. Definite diagnosis is made on biopsy of a specimen taken via flexible bronchoscopy, with the specimen positive for Congo Red staining [2]. Pleuropulmonary amyloidosis most commonly arises due to the deposition of monoclonal immunoglobulin light chains produced by plasma cells. In systemic forms of amyloid light chain (AL) amyloidosis, these proteins are synthesized in the bone marrow and subsequently deposited in pulmonary tissues, including the lungs and pleura [3]. In contrast, localized AL amyloidosis originates within the lung itself, with amyloid deposits confined to the respiratory system. Systemic pulmonary involvement is more frequently associated with lambda light chains, whereas localized variants such as nodular pulmonary amyloidosis and tracheobronchial amyloidosis are more commonly linked to kappa light chains [4].

## Case presentation

**Figure 1:**
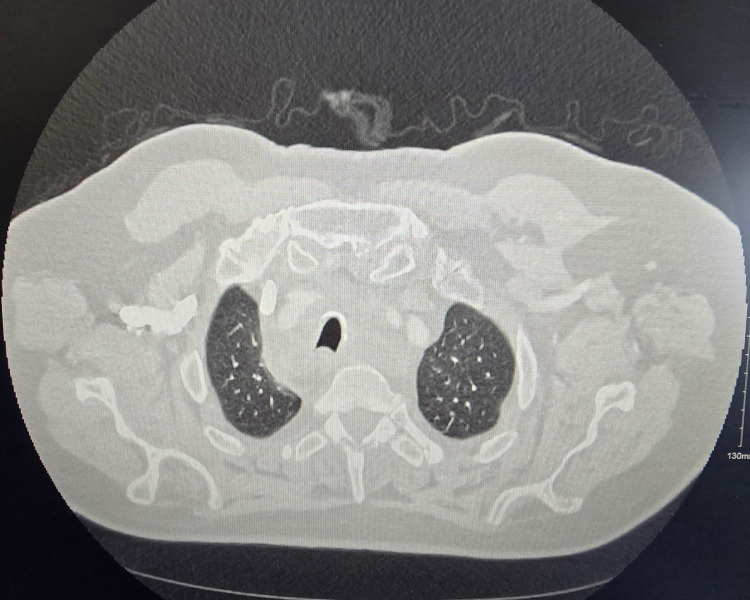
CT thorax with contrast showing slice at the level of the trachea

In 2024, a 71-year-old gentleman was referred to a tertiary care hospital complaining of worsening shortness of breath and dry cough. He was initially treated with antibiotics and inhalers with no improvement and was referred for a CT chest (Figure 1). CT scan showed extensive mediastinal and hilar lymphadenopathy. There was tracheal narrowing with high-grade stenosis of the left lower lobe bronchus with associated subtotal collapse of the left lower lobe. CT abdomen and pelvis showed pathologically enlarged enhancing lymph nodes noted in the superficial inguinal region bilaterally.

**Figure 2:**
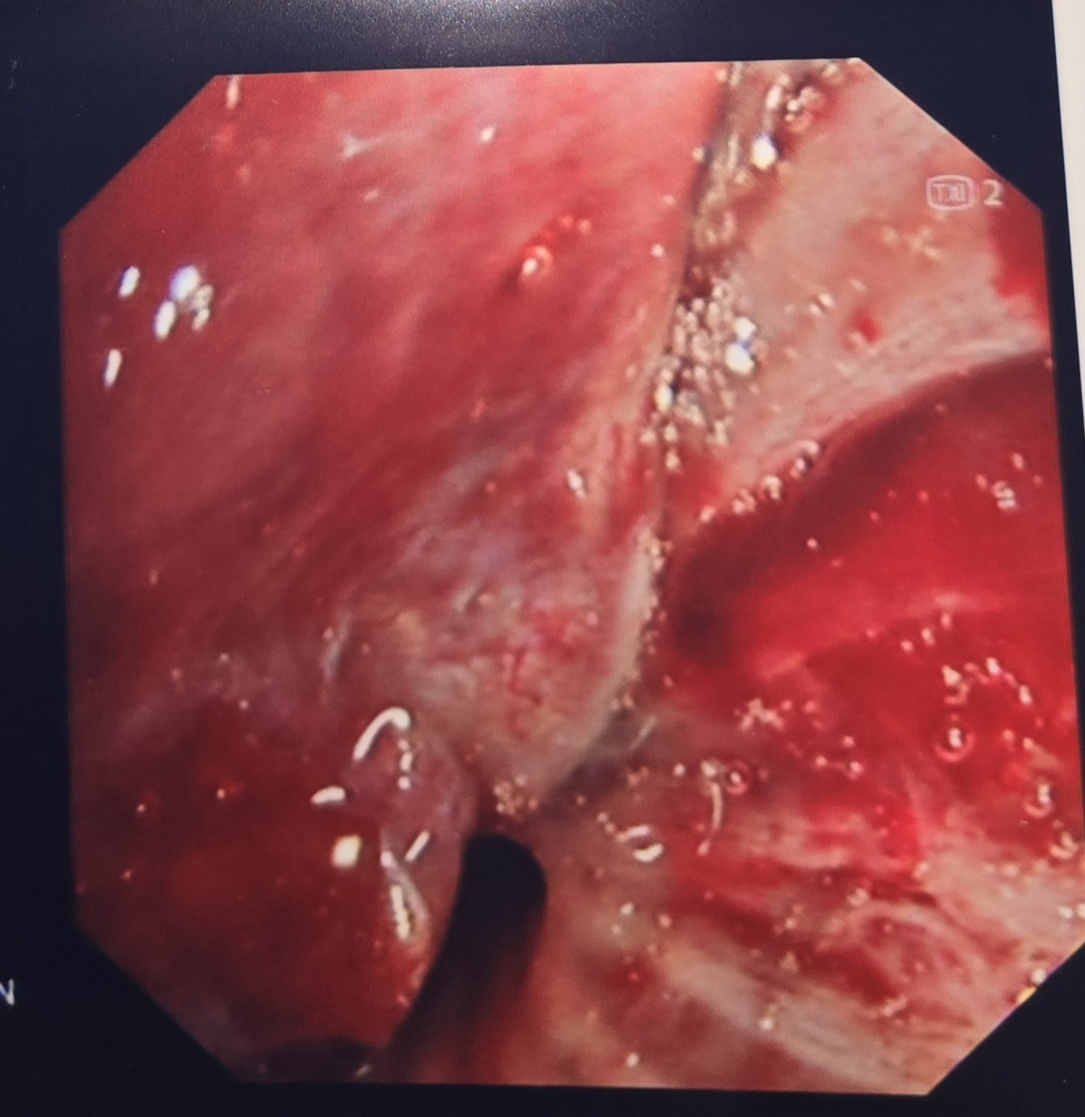
Endoscopic ultrasound (EBUS) image of the trachea showing narrowing

**Figure 3:**
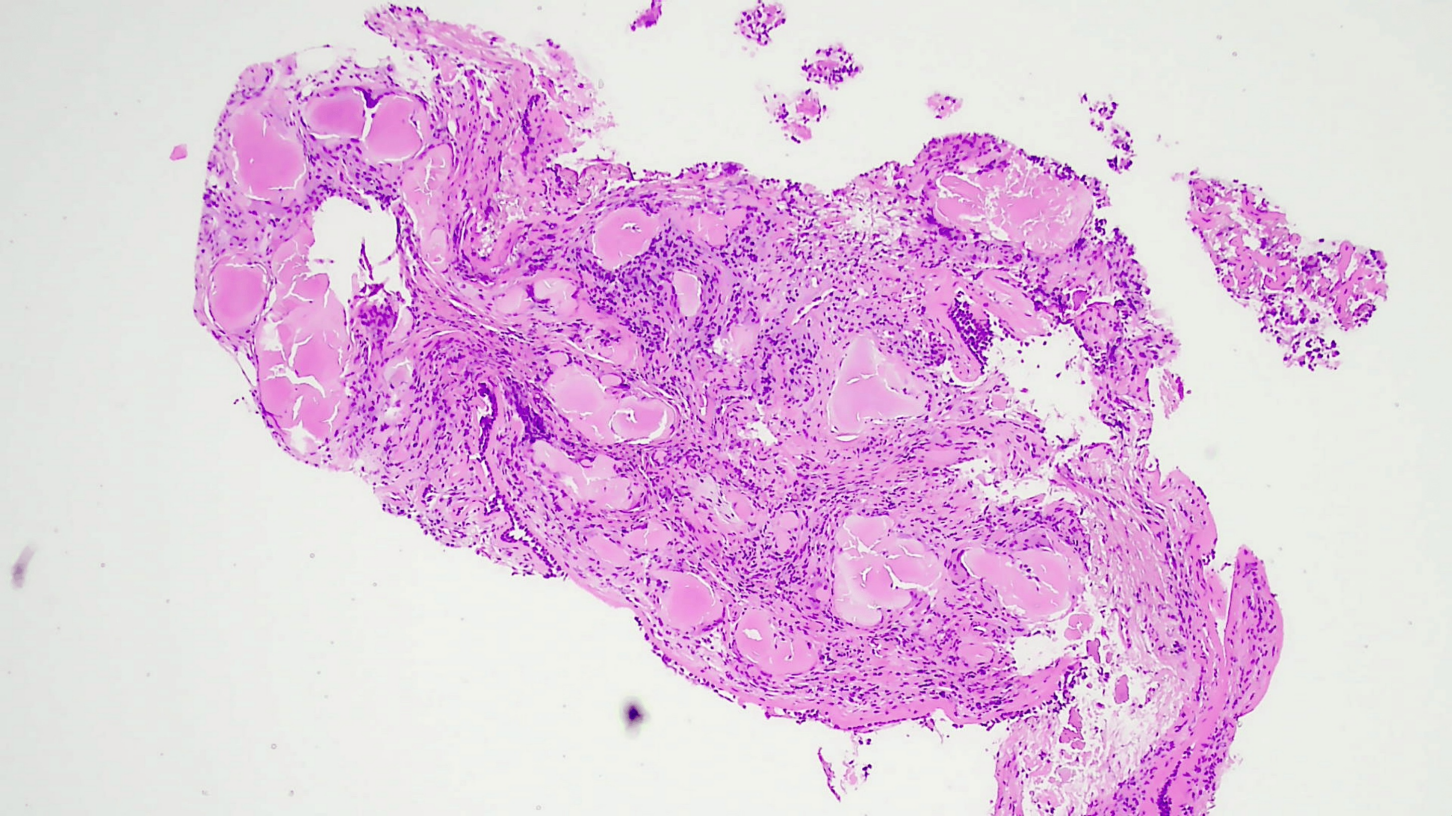
Histological section of the trachea showing amyloid deposition

**Figure 4:**
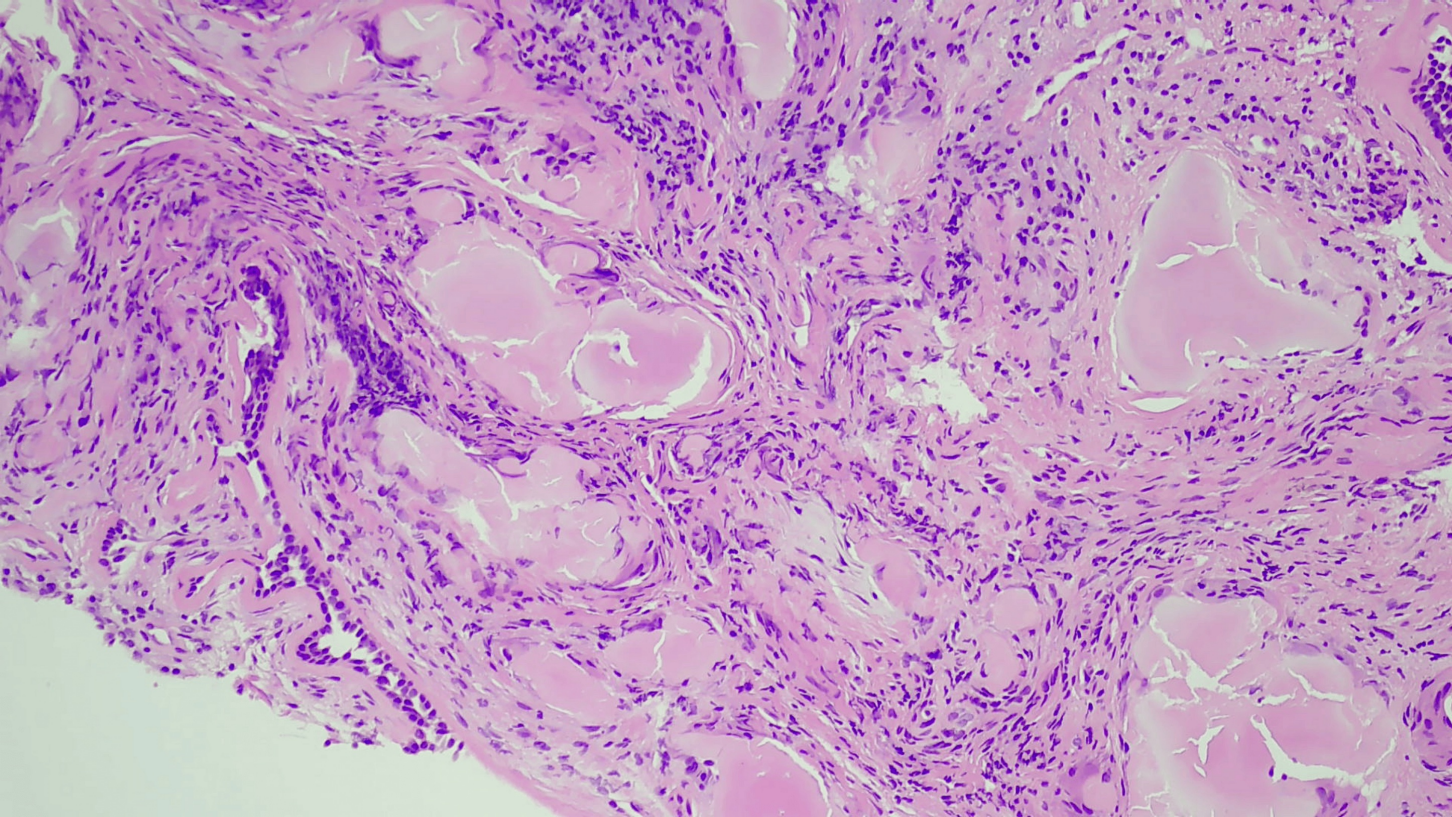
High-resolution histological image of the trachea showing amyloid deposition

**Figure 5:**
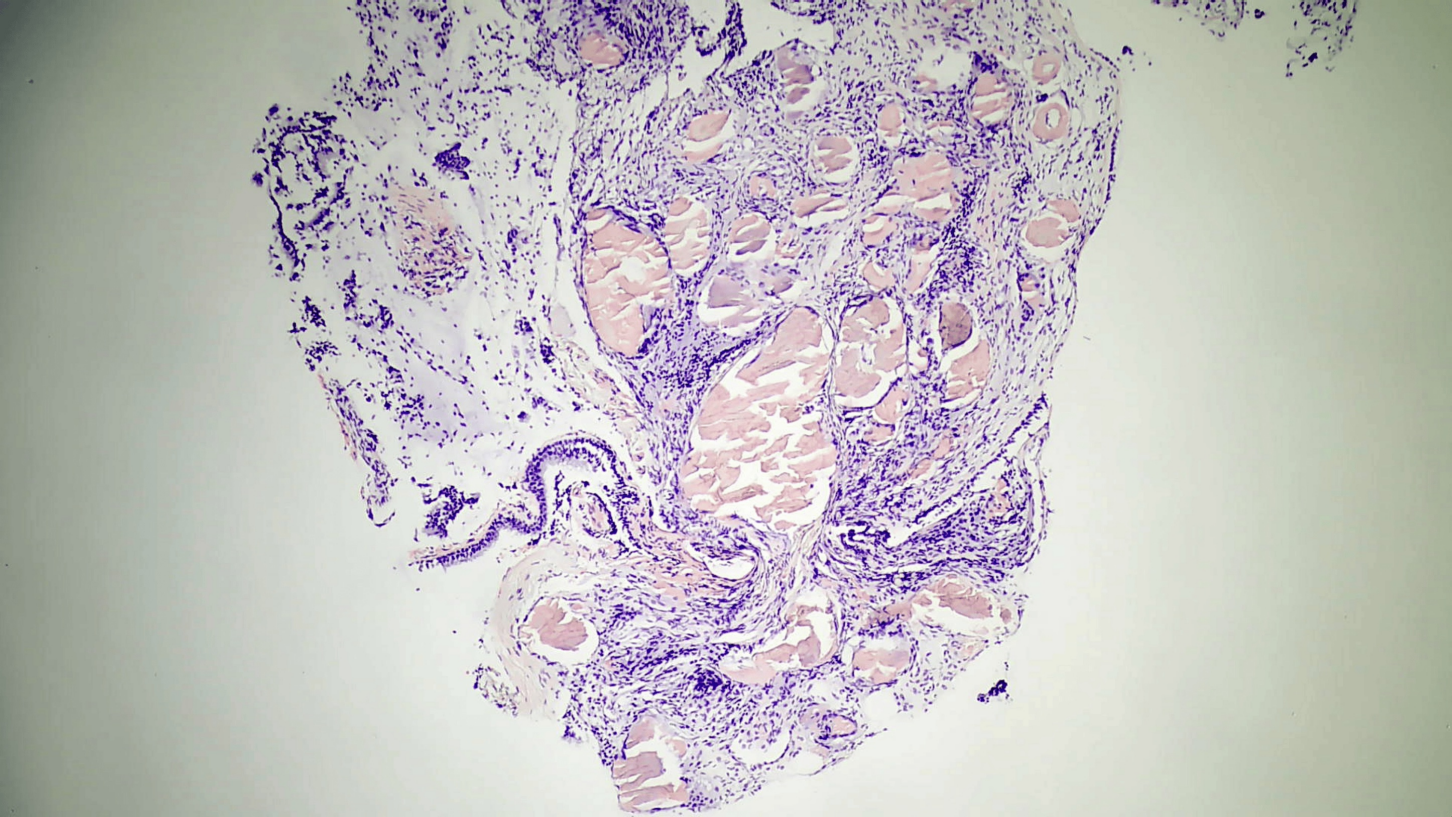
Congo red stain-positive amyloid deposition in the histological section

**Figure 6:**
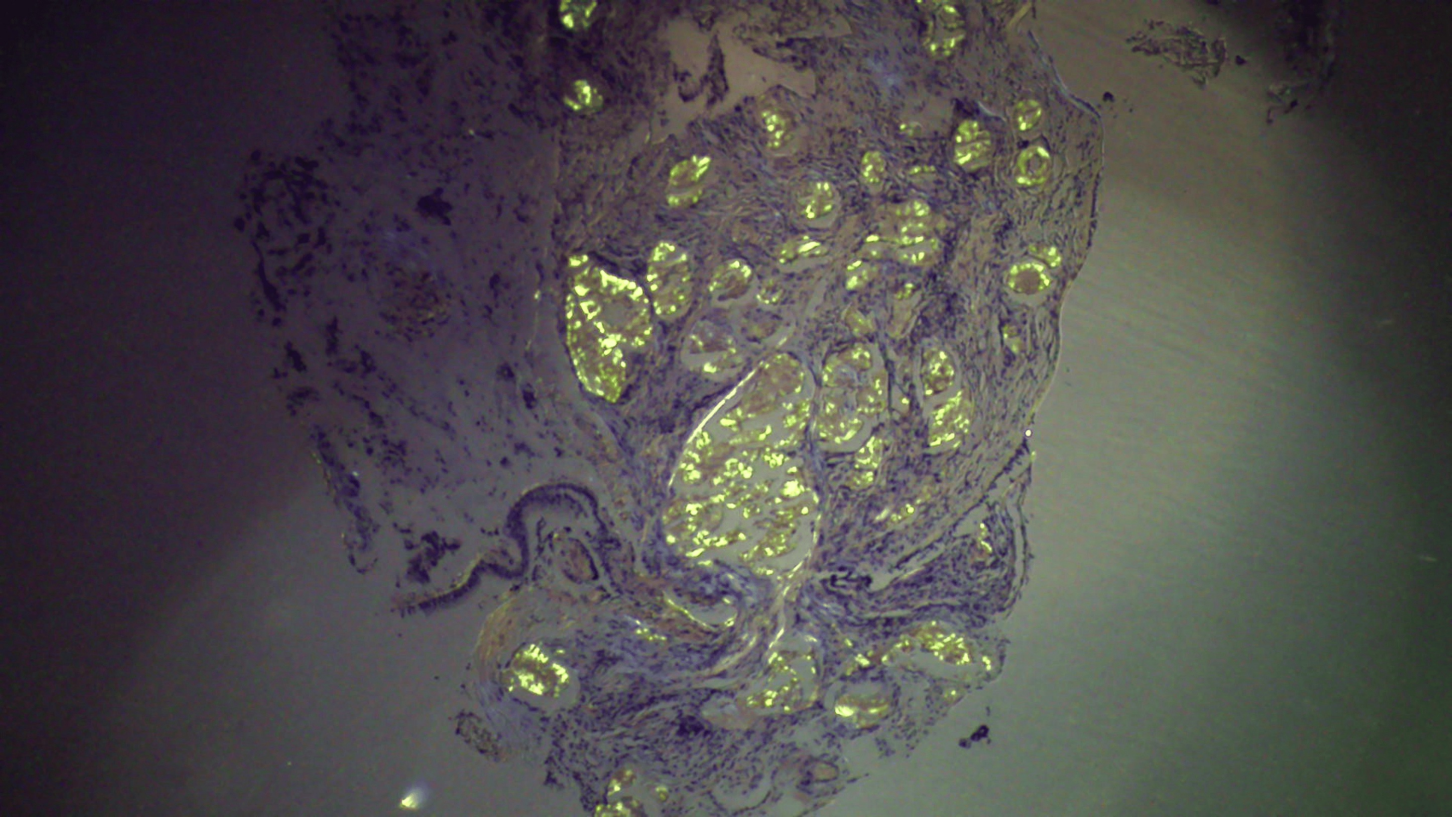
Typical Applegreen birefringence of amyloid deposit stained by Congo red in histological section

After reviewing the CT results, as part of the further workup, we planned an endobronchial ultrasound (EBUS). An EBUS is a procedure that uses an ultrasound with a bronchoscope to visualize and take biopsies within the lung. EBUS showed tracheal narrowing with endobronchial lesion in the left tracheobronchial tree, partially obstructing the left lower lobe bronchus (Figure 2). The biopsy results taken from the proximal trachea reported bronchial mucosa showing deposition of nodular acellular Congo red material with Applegreen birefringence on fluoride NG light consistent with amyloid (Figures 3-6).

The patient had an excisional biopsy of one of the inguinal lymph nodes. The biopsy results suggested a low-grade lymphoproliferative disorder with marked plasmacytic differentiation contributing to AL amyloid. He is currently under the care of a Consultant Hematologist for treatment of lymphoma with bortezomib, rituximab, and dexamethasone. 

Histopathological features

Tracheobronchial Tree

Bronchial mucosa showed deposition of the nodular, acellular, congophilic material with Applegreen birefringence on a polarizing light, consistent with amyloid (Figures 3-6).

A bronchoalveolar lavage (BAL) from the left lower lobe revealed benign cytology with mild acute inflammation. Brushings obtained from the same lobe also showed benign cytology. An EBUS transbronchial needle aspiration (TBNA) from station 11R was inadequate and nondiagnostic. However, EBUS TBNA from station 4R demonstrated benign lymph node content. Samples from stations 4L and 7 were both inadequate and nondiagnostic.

## Discussion

Amyloidosis is characterized by the misfolding of specific proteins, leading to their aggregation into insoluble fibrils that deposit in tissues and disrupt normal organ function. Although more than 30 proteins are known to have amyloid-forming potential, only a limited subset is implicated in pulmonary involvement. Among the systemic forms of amyloidosis, immunoglobulin AL and transthyretin (ATTR) amyloidosis are the most commonly associated with thoracic manifestations of the disease [5].

Types of amyloid proteins involving the pleuropulmonary system

Several types of amyloid proteins can contribute to pleuropulmonary amyloidosis, each with distinct patterns of organ involvement and clinical presentation. Among them, immunoglobulin AL amyloidosis is the most prevalent type affecting the lungs and pleura. AL amyloid fibrils are composed of either lambda or kappa light chains. In systemic AL amyloidosis, which often leads to multiorgan involvement, including the lungs, lambda light chains are predominant and account for approximately two-thirds of cases. Conversely, in localized AL amyloidosis affecting the respiratory tract, such as nodular pulmonary amyloidosis or tracheobronchial amyloid disease, kappa light chains are more commonly identified.

Another important but less frequent contributor to pleuropulmonary amyloidosis is ATTR amyloidosis. ATTR-related amyloid deposits are primarily known for their role in cardiac and neuropathic disease, but can occasionally present with thoracic involvement. ATTR amyloidosis exists in two forms: wild-type ATTR (ATTRwt), which is associated with aging and occurs sporadically, and variant or hereditary ATTR (ATTRm), caused by mutations in the ATTR gene. While pulmonary involvement is rare in both forms, it may occasionally present as part of a broader systemic disease picture. Understanding the biochemical composition and clinical behavior of amyloid subtypes is crucial in determining the extent of thoracic disease and guiding appropriate diagnostic and therapeutic strategies [6].

Secondary, or amyloid A (AA) amyloidosis, arises from the deposition of serum amyloid A (SAA) protein, often in association with chronic inflammatory conditions. In some cases, amyloid formation may also involve apolipoprotein variants, specifically AApoAI and AApoAII. Although AA amyloidosis is well-recognized for its involvement in organs such as the kidneys and liver, it rarely presents with clinical signs of respiratory involvement, making pulmonary manifestations exceedingly uncommon in this subtype.

In contrast, the majority of pulmonary amyloidosis cases are linked to AL amyloidosis, which is driven by plasma cell dyscrasias. In this setting, monoclonal free light chains, typically either lambda or kappa, are produced by abnormal plasma cells. These misfolded proteins then aggregate and deposit as amyloid fibrils. When the light chains originate in the bone marrow and circulate systemically before being deposited in the lung, the condition is classified as systemic AL amyloidosis. Alternatively, when these light chains are produced and deposited locally within the lung tissue, the disease is referred to as localized AL amyloidosis. This form may involve the lung parenchyma, airways, or pleura, and typically progresses at a slower rate with a more indolent clinical course compared to systemic involvement. Understanding the distinct subtypes and mechanisms of amyloid deposition is essential for accurate diagnosis and tailored management of pleuropulmonary amyloidosis [7,8].

Tracheal amyloidosis contributes to 1% of benign tumors of the tracheobronchial tree [9]. The incidence of immunoglobulin light chain amyloidosis is 8-12 persons per million per year [10]. Localized amyloidosis represents about 10% of all amyloidosis cases. A definitive diagnosis is typically established through the observation of birefringence in Congo red-stained tissue when examined under polarized light microscopy.

## Conclusions

Tracheal amyloidosis is an uncommon condition marked by the accumulation of misfolded proteins in the tracheobronchial tree, accounting for roughly 1% of benign tumors in this area. Immunoglobulin light chain amyloidosis has an estimated incidence of 8-12 cases per million people annually. The localized form of amyloidosis represents about 10% of all cases, and its diagnosis is confirmed by observing birefringence in Congo red-stained tissue under polarized light microscopy. This case report underscores a typical presentation of a rare respiratory disorder. Due to its nonspecific symptoms, diagnosing tracheal amyloidosis can be difficult, which may lead to delays in proper treatment. In these instances, bronchoscopy serves as an important diagnostic tool, helping to establish a clear diagnosis and rule out other, more common lung conditions. Once the diagnosis is confirmed, it is crucial to exclude any secondary causes of amyloidosis. A collaborative, multidisciplinary approach is vital for effective management, and ongoing follow-up is essential to monitor the patient’s condition and promptly address any emerging complications.
